# Compound Cytotoxicity Profiling Using Quantitative High-Throughput Screening

**DOI:** 10.1289/ehp.10727

**Published:** 2007-11-22

**Authors:** Menghang Xia, Ruili Huang, Kristine L. Witt, Noel Southall, Jennifer Fostel, Ming-Hsuang Cho, Ajit Jadhav, Cynthia S. Smith, James Inglese, Christopher J. Portier, Raymond R. Tice, Christopher P. Austin

**Affiliations:** 1 NIH Chemical Genomics Center, National Institutes of Health, Department of Health and Human Services, Bethesda, Maryland, USA; 2 National Toxicology Program and; 3 National Center for Toxicogenomics, National Institute of Environmental Health Sciences, National Institutes of Health, Department of Health and Human Services, Research Triangle Park, North Carolina, USA

**Keywords:** 1,536-well, cell viability, NTP 1,408 compound library, PubChem, qHTS, RT-CES

## Abstract

**Background:**

The propensity of compounds to produce adverse health effects in humans is generally evaluated using animal-based test methods. Such methods can be relatively expensive, low-throughput, and associated with pain suffered by the treated animals. In addition, differences in species biology may confound extrapolation to human health effects.

**Objective:**

The National Toxicology Program and the National Institutes of Health Chemical Genomics Center are collaborating to identify a battery of cell-based screens to prioritize compounds for further toxicologic evaluation.

**Methods:**

A collection of 1,408 compounds previously tested in one or more traditional toxicologic assays were profiled for cytotoxicity using quantitative high-throughput screening (qHTS) in 13 human and rodent cell types derived from six common targets of xenobiotic toxicity (liver, blood, kidney, nerve, lung, skin). Selected cytotoxicants were further tested to define response kinetics.

**Results:**

qHTS of these compounds produced robust and reproducible results, which allowed cross-compound, cross-cell type, and cross-species comparisons. Some compounds were cytotoxic to all cell types at similar concentrations, whereas others exhibited species- or cell type–specific cytotoxicity. Closely related cell types and analogous cell types in human and rodent frequently showed different patterns of cytotoxicity. Some compounds inducing similar levels of cytotoxicity showed distinct time dependence in kinetic studies, consistent with known mechanisms of toxicity.

**Conclusions:**

The generation of high-quality cytotoxicity data on this large library of known compounds using qHTS demonstrates the potential of this methodology to profile a much broader array of assays and compounds, which, in aggregate, may be valuable for prioritizing compounds for further toxicologic evaluation, identifying compounds with particular mechanisms of action, and potentially predicting *in vivo* biological response.

Animal toxicity data is used to predict human toxicity, based on the assumption that adverse effects in laboratory animals indicate the potential for adverse effects in humans. Various animal models have been developed to evaluate a broad range of toxicologic responses in order to classify compounds by their potential for causing adverse health effects in humans. These animal models include acute, subchronic, and/or chronic tests for end points such as oral, dermal, and ocular toxicity; immunotoxicity; genotoxicity; reproductive and developmental toxicity; and carcinogenicity (Chhabra et al. 2003). Animal tests, while clearly useful, can be relatively expensive and low throughput. Furthermore, intrinsic differences in species sensitivity can confound the extrapolation of certain test results to human health effects. Also, there is increasing societal concern about the use of animals in testing, especially in test methods that might induce pain and suffering in the treated animals (e.g., ocular toxicity). Thus, there is increased interest among the international scientific community in the development, translation, validation, and use of nonanimal alternative test methods for making regulatory decisions [European Center for the Validation of Alternative Methods (ECVAM) 2007; Interagency Coordinating Committee on the Validation of Alternative Methods (ICCVAM) 2007; National Research Council 2007; Tweats et al. 2007]. Given these scientific and societal issues, and the increasing number of new compounds requiring toxicity testing, the National Toxicology Program (NTP) recently began a major initiative to develop a high-throughput screening (HTS) program to prioritize compounds for further in-depth toxicologic evaluation, identify mechanisms of action for further investigation, and develop predictive models for *in vivo* biological response (NTP 2004a; Tice et al. 2007).

In support of this initiative, the NTP and the National Institutes of Health (NIH) Chemical Genomics Center (NCGC) formed a partnership in 2005 to *a*) develop a library of compounds suitable for HTS that had been characterized to some degree by traditional toxicologic testing methods, *b*) identify and/or develop cell-based or biochemical HTS assays potentially informative for *in vivo* toxicologic effects, and *c* ) profile the compound library in these HTS assays. The ultimate goal of this collaboration is to establish *in vitro* “signatures” of *in vivo* rodent and human toxicity by comparing the data generated in HTS assays with the rich historical database generated by the NTP using traditional *in vivo* and *in vitro* toxicologic assays. The U.S. Environmental Protection Agency (EPA) has also recognized the potential of high-throughput screening in toxicology testing and has initiated the ToxCast program for prioritizing the toxicity testing of environmental chemicals (Dix et al. 2007).

HTS was developed by the pharmaceutical industry to evaluate the biological activity of thousands of chemicals to identify potential drug candidates. Because HTS for this purpose is generally performed at a single concentration only (typically 10 μM), the approach is characterized by a high prevalence of false positives and negatives. To address these limitations and make HTS useful for toxicology and chemical genomics, the NCGC developed the quantitative high-throughput screening (qHTS) paradigm (Inglese et al. 2006). With this approach, all compounds are screened for a concentration-dependent response, which allows for a more accurate assessment of biological activity.

Here, we report on the use of qHTS to profile the cytotoxicity (the term “cytotoxicity” is used to describe the cumulative effect of a compound over a given period of time on cell number, whether due to apoptosis, necrosis, or a reduction in the rate of cell proliferation) of 1,408 compounds in 13 cell types using a homogeneous, luminescent cell viability assay that measures the intracellular levels of adenosine triphosphate (ATP) as an indicator of the number of metabolically active cells. Selection of the 1,408 compounds was based in part on the availability of toxicologic data from standard tests for carcinogenicity, genotoxicity, immunotoxicity, and/or reproductive and developmental toxicity; all compounds were tested at 14 concentrations from 0.59 nM to 92 μM. The cell types used in this evaluation include corresponding human and rodent cells derived from six tissues (liver, blood, kidney, nerve, lung, skin) that are common targets of xenobiotic toxicity. Using this approach, we developed species- and cell type–specific cytotoxicity profiles for each compound. Furthermore, we demonstrate that compounds with similar end point toxicity may exhibit different cytotoxicity kinetics, suggestive of different mechanisms of action. *In vitro* profiling of compounds promises to provide information on molecular mechanisms of toxicity, and may allow the creation of algorithms for predictive *in vivo* toxicology.

## Materials and Methods

### The NTP 1,408 compound library

A collection of 1,408 substances (except where noted, the term “substance” is used interchangeably with “compound” here) was constructed for characterization in qHTS assays (Smith et al. 2007; Tice et al. 2007); 1,408 is the number of substances that can fit in a single 1,536-well plate exclusive of controls. To allow evaluation of assay reproducibility, 55 of the compounds were represented twice in the collection, giving a total of 1,353 unique compounds. Of these, 1,206 had been tested by the NTP in one or more *in vitro* and/or *in vivo* assays, including those for *Salmonella typhimurium* mutagenicity (68%), chronic toxicity/carcinogenicity (23%), reproductive toxicity (3%), developmental toxicity (3%), and immunotoxicity (1%). Also included were 147 reference compounds identified by the ICCVAM for the development and/or validation of alternative *in vitro* test methods for dermal corrosivity, acute toxicity, and endocrine activity. Molecular weights of all compounds ranged from approximately 32 (methanol) to 1,300 (actinomycin D), with 95% of the compounds having a molecular weight that was < 400. Functionally, the NTP library of 1,408 compounds includes solvents, fire retardants, preservatives, flavoring agents, plasticizers, therapeutic agents, inorganic and organic pollutants, drinking-water disinfection by-products, pesticides, and natural products. Compounds excluded from this NTP collection were those considered excessively volatile and those not soluble in dimethylsulfoxide (DMSO), the solvent used for compound transfer. A complete list of the NTP 1,408 compounds and full chemical descriptions are publicly available (PubChem 2007a).

All compounds were received from suppliers via the NTP chemistry support contract in 1-mL aliquots at 10 mM dissolved in DMSO and stored at −80°C in Matrix TrakMates 2D bar-coded storage tubes (Thermo Fisher Scientific, Hudson, NH). Purity and identity information for the compounds was obtained from the suppliers and, in the case of compounds used in NTP studies, from the characterizations performed in support of those studies. With the exception of natural compounds and other known mixtures, most compounds were > 90% pure.

Sets of compounds prepared as 10-mM stock solutions and stored in 96-well plates were compressed into 384-well plates. From these plates, fifteen 384-well plates containing the 1,408 compounds at 2.236-fold dilutions were prepared using an Evolution P^3^ system (PerkinElmer, Inc., Wellesley, MA). The sets of 384-well plates composing the dilution series were then compressed into multiple 1,536-well plates by interleaved quadrant transfer. During screening, working copies of the 1,536-well compound plates were stored at room temperature for up to 6 months; back-up copies were heat sealed and stored at −80°C.

### Cell types and culture conditions

Human embryonic kidney cells (HEK293), human hepatocellular carcinoma cells (HepG2), human neuroblastoma cells (SH-SY5Y and SK-N-SH), human leukemia T cells (Jurkat, clone E6-1), normal human foreskin fibroblasts (BJ), normal human lung fibroblasts (MRC-5), normal human vascular endothelial cells (HUVEC), rat hepatoma cells (H4-II-E), mouse neuroblastoma cells (N2a), and mouse fibroblast cells (NIH 3T3) were purchased from the American Type Culture Collection (ATCC, Manassas, VA). Human renal mesangial cells obtained from adult kidney tissue were kindly provided by J. Kopp (National Institute of Diabetes and Digestive and Kidney Diseases, NIH, Bethesda, MD). Rat renal proximal tubule cells were freshly isolated from rat kidney by In Vitro ADMET Laboratories, LLC (Rockville, MD). All cells chosen for testing represent target tissues of interest in toxicology from both human and rodent sources; most were transformed lines, but some were nontransformed or primary cells. All are well-characterized cells, produce uniform populations, are relatively easy to culture, and are amenable to a 1,536-well format. The origin and status of each cell type are presented in Table 1. We determined the doubling times for each cell line using a hemocytometer at designated intervals (McAteer and Davis 2002).

We cultured cells in ATCC complete Eagle’s minimal essential medium (N2a, H4-II-E, SK-N-SH, MRC-5, BJ, HepG2, and HEK293), ATCC complete Dulbecco’s minimal essential medium (DMEM) (NIH 3T3), RPMI 1640 (Jurkat and human renal mesangial cells), or ATCC complete DMEM/F-12 (SH-SY5Y and rat renal proximal tubule cells). These media were supplemented with 10% fetal bovine serum (Invitrogen, Carlsbad, CA), 50 U/mL penicillin, and 50 μg/mL streptomycin (Invitrogen). Human HUV-EC-C cells were cultured in ATCC Kaighn’s F12K medium supplemented with 0.1 mg/mL heparin (Sigma-Aldrich, St. Louis, MO), 0.04 mg/mL endothelial cell growth supplement (Sigma-Aldrich). Each cell type was maintained at 37°C under a humidified atmosphere and 5% CO_2_.

### Cell viability assay

We measured cell viability using a luciferase-coupled ATP quantitation assay (CellTiter-Glo; Promega, Madison, WI). In this assay, luminescent signal is proportional to amount of ATP and thus to the number of metabolically competent cells; cell injury and death result in a dramatic decrease in intracellular ATP levels (Crouch et al. 1993). Cells were dispensed at 1,000–2,000 cells/5 μL/well in tissue-culture treated 1,536-well white/solid bottom assay plates (Greiner Bio-One North America, Monroe, NC) using a Flying Reagent Dispenser (Aurora Discovery, Carlsbad, CA). All but Jurkat cells (which are grown in suspension) were incubated at 37°C for 5–6 hr to allow for cell attachment, followed by addition of compounds via pin tool (Kalypsys, San Diego, CA). After compound addition, plates were incubated for 40 hr at 37°C; incubation duration was based on results of assay optimization experiments (data not shown) and is the longest duration that can be used without evaporation-induced edge effects occurring in 1,536-well plates. At the end of the incubation period, 5 μL of CellTiter-Glo reagent was added, plates were incubated at room temperature for 30 min, and the luminescence intensity of each well was determined using a ViewLux plate reader (PerkinElmer, Shelton, CT).

To avoid false positive or false negative results due to luciferase inhibition or signal interference from optically active compounds, respectively, we tested all compounds in the presence of a physiologically relevant concentration of ATP in each well (data not shown). Of the 25 compounds that inhibited luciferase to any degree, the extent of luciferase inhibition was not sufficient to interfere with measurement of cytotoxicity.

### qHTS

Compound formatting and qHTS were performed as described previously (Inglese et al. 2006). Two positive control compounds were dispensed on each plate: doxorubicin in a concentration series from 0.7 nM to 100 μM in DMSO in column 1, and tamoxifen in concentration series from 0.23 to 100 μM in DMSO in column 2. DMSO only was dispensed into column 3, and tamoxifen at 100 μM in DMSO in column 4.

Transfer of 23 nL of experimental compounds was performed via pin tool (Cleveland and Koutz 2005), resulting in final concentrations of 0.59 nM–46 μM of compound, and 0.45% DMSO. To achieve a highest final compound concentration of 92 μM (DMSO concentration, 0.9%), 23 nL was transferred twice from the highest concentration mother plate into each well of the assay plate; control plates using DMSO only at this higher concentration were also included. DMSO tolerance experiments with each cell type showed no effect on viability at concentrations up to 1% (data not shown).

Inclusion of 55 duplicate compounds in the compound plate allowed for measurement of intra-experimental reproducibility in every assay at every concentration. To test inter-experimental reproducibility, qHTS of HepG2 cells was performed across the entire 1,408 collection 3 times, with one screen being done on each of 3 different weeks using identical assay conditions.

### Real-time cell electronic sensing (RT-CES) cytotoxicity assay

HepG2 cells in the presence of compounds at concentrations from 0.14 to 100 μM were monitored by measurements of electrical impedance (ACEA Biosciences Inc., San Diego, CA) every 10 min for the first hour, then every hour for 70–90 hr. Continuous recording of impedance in cells was reflected by cell index value (Xing et al. 2005).

### Data analysis and curve fitting

Analysis of compound concentration–response data was performed as previously described (Inglese et al. 2006). Raw plate reads for each titration point were first normalized relative to the tamoxifen control (100 μM, 100%) and DMSO-only wells (basal, 0%), then corrected by applying a pattern correction algorithm using compound-free control plates (i.e., DMSO-only plates) at the beginning and end of the compound plate stack. Concentration–response titration points for each compound were fitted to the Hill equation (Hill 1910), yielding concentrations of half-maximal inhibition (IC_50_) and maximal response (efficacy) values. Compounds were designated as classes 1–4 according to the type of concentration–response curve observed (Inglese et al. 2006): Class 4 compounds show no concentration response or have no significant activity point, that is, ≥ 3 SD of the activity at the lowest concentration tested; class 3 compounds display significant activity only at the highest concentration tested; class 2 compounds have incomplete curves (i.e., no low-concentration asymptote) and class 1 compounds have complete response curves (i.e., two asymptotes). Class 1 or 2 compounds were further divided into subclasses based on efficacy and quality of fit (*R*^2^). Compounds with *R*^2^ > 0.9 and high (> 80%) efficacy were designated as subclass a, and compounds with *R*^2^ > 0.9 and low efficacies (30–80%) as subclass b. Compounds with class 1a, 1b or 2a curves were generally selected for follow-up analyses as they represented high-confidence data. Compound activity correlations between different assays or experiments were assessed by calculating the Pearson correlation coefficient. Hierarchical clustering of compound activity patterns across different assays was performed within Spotfire DecisionSite 8.2 (Spotfire Inc., Cambridge, MA) using the correlation of log IC_50_ values as the similarity metric. All the normalized cytotoxicity data obtained for the 1,408 compounds tested in the 13 cell types have been deposited into PubChem (http://www.ncbi.nlm.nih.gov/sites/entrez?db=pcassay, search term “NCGC [sourcename] AND Viability [AssayName]”) (PubChem 2007b).

## Results

A total of 1,408 known compounds previously studied by the NTP were successfully arrayed in 1,536-well plates in uniform solvent and concentrations. A luminescent cell viability assay was selected to investigate the feasibility of using 14-concentration qHTS for *in vitro* toxicity characterization of these compounds and was easily adapted to a 1,536-well format. Robust and reproducible data were obtained on the NTP compound library in this cytotoxicity assay. These data demonstrate the utility of the qHTS approach to toxicity investigations and will facilitate interpretation of data from subsequent cell-based qHTS assays conducted at NCGC on the NTP compound collection.

### Establishment of a positive control for cytotoxicity

qHTS is optimally informative when results are compared to a positive control. Establishment of a universal positive control for these studies was challenging because toxicity was to be tested across a wide variety of cell types. Initial testing of the 1,408 compounds identified two, tamoxifen and doxorubicin, that were toxic in all cell types and were therefore included as positive controls on all assay plates. Tamoxifen was used as the control to normalize response unless otherwise noted. Tamoxifen concentration–response curves were very consistent among plates within a cell type although potency was cell-type specific, with Jurkat cells being the most sensitive (IC_50_ = 8.3 μM), and NIH3T3, HEK293, BJ, and mesangial cells least sensitive (IC_50_ = 79 μM). The rank order of potency among the 13 lines is presented in Table 2. Z′-factors (Zhang et al. 1999) were between 0.44 and 0.91, and coefficients of variation (CV) were between 6.9 and 12.4, based on 234 plates used to test 1,408 compounds in 13 cell types (Table 2). Both sets of Z-factors and CV values indicate that the assays under the qHTS platform are very robust and suitable for accurately identifying cytotoxic compounds.

### Assay reproducibility

**Figure 1:**
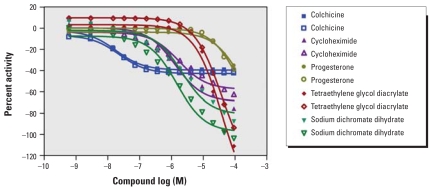
Intraexperiment reproducibility. The figure shows example replicate dose–response curves for colchicine, cycloheximide, progesterone, tetraethylene glycol diacrylate, and sodium dichromate dehydrate in rat primary kidney proximal tubule cells.

To evaluate intra-experimental reproducibility, we compared results for the 55 compounds present in duplicates in the collection. The correlation of IC_50_ for these replicates in all 13 cell lines was 0.71 (*p* < 0.001). In rat renal proximal tubule cells, the IC_50_ values for the 11 duplicate compounds that displayed a concentration–response relationship showed an excellent correlation (*R*^2^ = 0.97); all compounds cytotoxic in one replicate were cytotoxic in the other, and the same was true for noncytotoxic compounds. Examples of concentration–response curves for duplicate cytotoxic compounds in rat renal proximal tubule cells are shown in Figure 1.

To evaluate interexperimental reproducibility, we performed qHTS on the 1,408 compounds in HepG2 cells once per week for 3 weeks. The concentration–response relationship of each compound was classified (see details in “Materials and Methods”). The outcome for each compound in each weekly iteration was designated as cytotoxic (classes 1a, 1b, and 2a compounds), not cytotoxic (class 4) or inconclusive (other curve classes). Of the 1,353 unique compounds, 90% of the compounds had the same outcome in all three runs; 9.3% of the compounds had the same outcome in two runs only; and 0.7% of the compounds had different outcomes in all three runs. Of the 116 compounds that were cytotoxic in at least one run, 102 (87.9%) were cytotoxic in all three runs. The regression correlations of the IC_50_ values for the three independent runs were calculated (IC_50_ value for nontoxic compounds was set to 92 μM, the maximum concentration used in all experiments) and good linear correlations were observed, with an average correlation coefficient (*R*^2^) of 0.74 (*p* < 0.001; *n* > 1000).

### Cytotoxicity of the 1,408 NTP compounds

**Figure 2:**
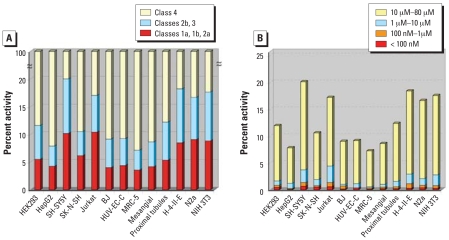
Pharmacological profile of compound activity. (*A*) Percentage of activity in each class identified from all compounds in 13 cell lines. (*B*) Potency distribution of all compounds in 13 cell lines.

Concentration–response relationship classification, potency, efficacy, and Hill coefficient were determined for all compounds and used to evaluate cytotoxicity of each compound in the 13 cell types tested. Of the 1,353 compounds tested, 428 showed cytotoxicity in at least one cell type. Results in rat primary kidney proximal tubule cells are presented to illustrate the type of data obtained for each cell type (Table 3). Seventy-nine compounds (6% of the total) produced class 1a, 1b, or 2a curves; these represent the highest confidence data for cytotoxicity. Another 96 (7%) produced class 2b or 3 curves, which represent lower confidence data for cytotoxic activity. The remaining 1,233 compounds (87%) did not induce a concentration-related increase in cytotoxicity and were classified as class 4. By comparing the percentage of compounds that produced class 1a, 1b, or 2a curves (percentages of all class 1–3 compounds are shown in parentheses) in each of the cell types (Figure 2A), a rank order of sensitivity was derived: Jurkat: 10.6% (17.2%) ≈ SH-SY5Y: 10.5% (20.1%) > N2a: 9.3% (17.0%) ≈ NIH 3T3: 9.1% (17.6%) > H-4-II-E: 8.5% (18.4%) > SK-N-SH: 6.4% (10.6%) > HEK293: 5.7% (11.9%) ≈ rat primary proximal tubule: 5.5% (12.4%) > HUV-EC-C: 4.5% (9.4%) ≈ mesangial: 4.3% (8.7%) ≈ HepG2: 4.3% (7.8%) ≈ BJ: 4.0% (9.1%) ≈ MRC-5: 3.8% (7.2%).

The IC_50_ values of the cytotoxic compounds ranged from double-digit nM to 80 μM (Figure 2B). For example, in rat primary kidney proximal tubule cells, three compounds had IC_50_ values <100 nM, 5 had values between 100 nM and 1 μM, 10 had values between 1 and 10 μM, and 60 had values between 10 and 80 μM (Table 3).

### Compound profiles across cell types

We used the differential cytotoxicity of compounds across cell types and species to construct profiles of cell lines according to the compounds that were toxic to them and profiles of compounds according to the cell lines in which they were toxic.

**Figure 3:**
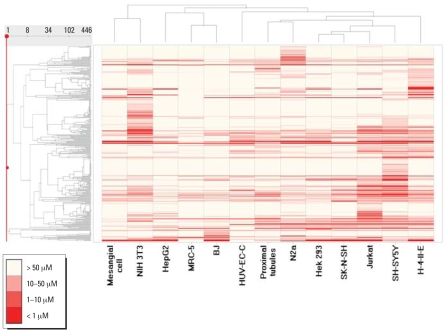
Compound activity patterns clustered by cell and species type. The compounds with an IC_50_ of < 92 μM in at least one cell type are selected and arranged in the order of a hierarchical clustering based on their IC_50_ values as shown in the dendrogram on the left side of the heat map. Neighboring compounds share similar activity patterns. In the figure, each row represents a compound, and each column is a cell type. Compound activity in each cell line is colored according to potency (IC_50_) range. Potent compounds are deeper shades of red and nontoxic compounds are white. Assays are also clustered by similarity in their compound IC_50_ patterns as shown in the dendrogram on the top of the heat map.

**Figure 4:**
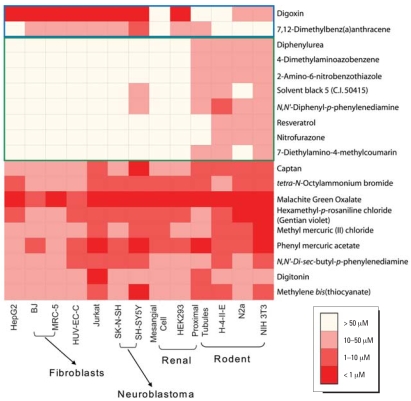
Compound activity across different species. Compound activity in each cell line is colored according to potency (IC_50_) range. The figure shows examples of compounds that are more cytotoxic to human cells (top two rows) or to rodent cells (middle rows), and compounds with similar levels of cytotoxicity in human and rodent cells (bottom rows).

To create compound profiles, compounds that demonstrated activity in at least one cell type were clustered by their IC_50_ values (Figure 3) across the 13 cell types. Some compounds, such as digitonin and the three rosaniline derivatives (hexamethyl-*p*-rosaniline chloride, pararosaniline hydrochloride, and malachite green oxalate) produced cytotoxicity in all cell types at similar concentrations (Figure 4). Other compounds, such as tetra-methylthiuram disulfide and methyl mercuric (II) chloride, were found to be selectively toxic to a particular cell type. For example, tetra-methylthiuram disulfide is 250-fold more potent in SH-SY5Y than the next most responsive cell type, and methyl mercuric (II) chloride is 50-fold more potent in NIH 3T3 than the next most responsive cell type.

Comparing species, some compounds such as zinc pyrithione showed similar toxicity in analogous human and rodent cell types, while others such as diphenylurea showed toxicity in cells from one species but not the other. In addition to compounds with differential toxicity between particular cell types in the two species, we were able to identify compounds that were generally cytotoxic either toward rodent or human cell types (determined by a *t*-test on IC_50_ values of the nine human versus the four rodent cell types). Thirty-nine compounds showed significant species selectivity (*p* < 0.05), 34 with greater potency in the rodent cells, and 5 with greater potency in human cells. For example, dimethylaminoazobenzene, a rodent liver carcinogen (Biswas and Khuda-Bukhsh 2005), was more toxic to rodent cells while digoxin, a Na^+^, K^+^-ATPase inhibitor (Sweadner 1989), was more toxic to human cells. Similar results for digoxin have been noted previously (Hengstler 1999), and other rodent-selective compounds have also been identified (Klaunig 2003).

### Cell-type profiles defined by compound responses

Similar to what was observed for cross-species comparisons, a range of relatedness was seen in the comparison of cell types. In some cases of closely related cell lines, such as MRC-5 and BJ human fibroblasts, the pattern of compound activity was quite similar (compare columns MRC-5 and BJ in heat map in Figure 3). However, in other closely related cell lines, such as the SK-N-SH and SH-SY5Y neuroblastoma cells (the former is the parental line of the latter), activity patterns were quite different (compare columns SK-N-SH and SH-SY5Y in heat map in Figure 3). Homologous cell types from different species produced patterns that were less related than those from nonhomologous cell types from the same species (compare columns SH-SY5Y and N2a, and HepG2 and H-4-IIE in heat map in Figure 3). For example, the human SH-SY5Y and rat N2a neuroblastoma cell lines produced quite different response patterns, as did the human HepG2 and rat H-4-II-E hepatoma lines. In general, rodent cells were more sensitive than human cells: on average, 6% of the compounds were designated as active (class 1a, 1b, and 2a) against human cells, compared to 8% active against rodent cells (Figure 2A). This difference is statistically significant (*p* < 0.001).

### Structure–activity relationships

As the 1,353 compounds were chosen on the basis of pre-existing toxicity data rather than structural relatedness, this modest set of compounds was too diverse to rigorously define any structure–activity relationships. However, we did observe that some structurally related compounds induced similar levels of cytotoxicity across cell types. For example, the three thiopurines—azathioprine, 6-mercaptopurine monohydrate, and 6-thioguanine—and the two organic mercury compounds—phenyl mercuric acetate and methyl mercuric (II) chloride—showed similar toxicity profiles across the 13 cell types and were nearest neighbors in the hierarchical clustering of activities. Data for a larger set of compounds with structurally related series are required to develop robust structural models of cytotoxicity or differential toxicity between cell types.

### Kinetic signatures of toxic compounds

**Figure 5:**
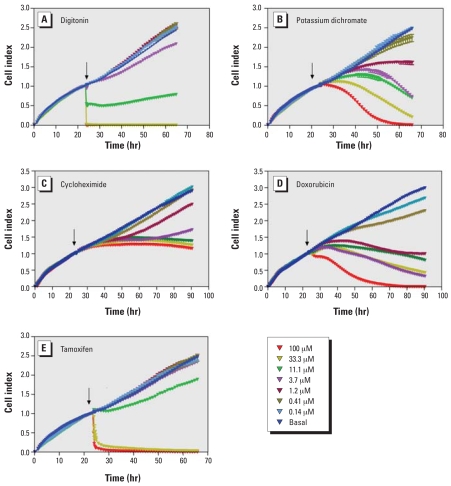
Kinetics of cytotoxicity responses for digitonin (*A*), potassium dichromate (*B*), cycloheximide (*C*), doxorubicin (*D*), and tamoxifen (*E*) in HepG2 cells monitored by the RT-CES system. 10,000 cells/well were plated in 16-well strips for the RT-CES cytotoxicity assay.↓, time of compound addition. Different compound concentrations are indicated by different colors. Data are normalized to the time of compound addition at 22–24 hr of cell culture.

To further characterize selected compounds identified in the end point cytotoxicity assay, cells exposed to selected compounds were dynamically monitored over 70 hr using real-time electrical impedance as a measure of viable cell number (Xing et al. 2005). Using this methodology, five well-characterized cytotoxic compounds were found to have different response kinetics. Over the concentration range tested, digitonin, potassium dichromate, doxorubicin, and tamoxifen were cytotoxic in all 13 cell types, and cycloheximide was cytotoxic in all but two cell types (MRC-5 and SH-SY5Y). However, time-course measurement of cytotoxicity in HepG2 cells, where all five compounds were cytotoxic, demonstrated distinct kinetic profiles that fit into three categories: acute (< 1 hr to full toxicity), subacute (1–40 hr), and long term (> 40 hr). Digitonin (Figure 5A), a mild detergent (Schulz 1990), and tamoxifen (Figure 5E), a Ca^2+^ influx stimulator, were fully cytotoxic to HepG2 cells after only 10 min of exposure. Potassium dichromate (Figure 5B) and doxorubicin (Figure 5D), both DNA-damaging agents (De Flora et al. 1990; Yang and Wang 1999), demonstrated a slower onset of activity and induced complete cytotoxicity only after 35 hr of exposure. Finally, cycloheximide (Figure 5C), a protein synthesis blocker (Liu et al. 1994), inhibited cell proliferation but did not decrease cell number below what was present at the time of addition.

## Discussion

More than 80,000 chemical compounds are registered for use in the United States (NTP 2004b). In addition, about 2,000 new compounds are introduced into commercial use each year that may pose hazards for human health (NTP 2004b). Traditional toxicologic methods cannot characterize and define the toxicity of such a large number of compounds in a cost-efficient and timely manner. However, recently developed HTS technologies may help to solve this problem by identifying classes of compounds with similar activity profiles and by helping to select and prioritize which compounds should receive a comprehensive toxicologic evaluation. As a first step in its HTS initiative, the NTP selected 1,353 compounds for characterization in cell-based cytotoxicity screens. The choice of compounds for this initial screening set was based primarily on practical and operational considerations. For example, the compounds had to be readily commercially available, of adequate purity and quantity, and soluble in DMSO up to the maximum stock concentration of 10 mM. This, and the fact that the entire universe of compounds of potential toxicologic interest stretches into the tens of thousands, means that many important compounds were not included in this initial set of 1,353. Now that proof of principle has been established in this robust and reproducible cell-based cytotoxicity assay, the compound collection is being expanded to include a greater number and diversity of compounds.

The human and rodent primary cells and cell lines used in the study were selected to represent six tissues of interest in toxicology: liver, blood, kidney, nerve, lung, and skin. Nine different cell types from human and four from rodents were chosen to gather data on potentially varied organ- and tissue-specific responses to environmental agents. Because our goal with this study was to investigate the feasibility of performing high-throughput assays of toxicologic compounds, we conducted our initial experiments with cell lines commonly used in qHTS: HepG2, SH-SY5Y, HEK293, Jurkat, H-4-II-E, and N2a (Hynes et al. 2006; Knasmuller et al. 2004). These lines are transformed or otherwise adapted to growing *in vitro*, so presumably are less representative of *in vivo* responses than primary cells, but they served to establish the qHTS methods used here and provided a baseline against which future studies with primary cells will be compared. In addition, several types of nontransformed and primary cells were used in this study, including human umbilical vein endothelial capillary cells (HUVECs) (Hoshi and Mckeehan 1984), MRC-5 normal human fetal lung fibroblasts (Jacobs et al. 1970), BJ normal human foreskin fibroblasts (Steinert et al. 2000), human kidney glomerular mesangial cells, and primary rat renal proximal tubule cells. The ability to use these cells in these qHTS assays suggests that future efforts in toxicologic profiling using primary cells will be feasible.

Reproducibility of qHTS data has been demonstrated previously (Inglese et al. 2006), but given the particular importance of reliability in toxicity profiling, we performed additional studies of assay robustness and reproducibility both within an assay and between replicates of an assay over time. In addition we evaluated the concentration–response curves for the positive controls included in each plate and found them to be very consistent. IC_50_ values of active compounds among 55 duplicates included in the compound library exhibited excellent correlation (*R*^2^ = 0.71; *R*^2^ = 0.89 when compounds with efficacies lower than 30% were removed from the analysis), demonstrating high intra-experiment reproducibility. To assess interassay reproducibility, three independent runs of the HepG2 qHTS were compared, producing a good correlation and average *R*^2^ of 0.74. Taken together, these data demonstrate that the ATP-based cytotoxicity assay in the qHTS platform is highly reproducible.

Of the 1,353 unique compounds tested, only 428 produced a measurable response in the cytotoxicity assays. Although this activity rate is higher than the expected rate in high-throughput screens for drug discovery, these results represent the prevalence of cytotoxicity at 40 hr of exposure in these cell types, measured with this readout, under these conditions at these concentrations, in the absence of metabolism. Many of the 1,353 compounds tested have been associated only with more chronic or subtle toxicity *in vivo*, so only a small subset of the compounds might reasonably be expected to be positive in this measure of acute cell killing.

Within the subgroup of 428 cytotoxic compounds, we were able to identify multiple patterns of effects within and across compound types, cell types, and species. Some compounds (e.g., digitonin, phenyl mercuric acetate) were uniformly toxic across all cell types, whereas others showed selective toxicity (e.g., 2-methyl-1-nitroanthraquinone in HEK293 cells). To the degree that structurally related compounds were present in this limited collection, nascent structure–activity relationships could sometimes be detected (e.g., the organic mercurials). These results suggest that a full range of similarities and differences in compound effects are potentially detectable using cell-based qHTS.

A range of cytotoxicity response patterns was also seen among cell types. Overall, the human blood- (Jurkat) and neuron-derived (SH-SY5Y) cells and rodent cells (N2a, H-4-II-E and NIH 3T3) were most sensitive to compound-induced cytotoxicity; kidney-derived cells (HEK293, human mesangial, and rat primary proximal tubule) were inter-mediate in sensitivity; and human fibroblastic, endothelial, and skin cells (HUV-EC-C, BJ, and MRC-5) were least sensitive. However, there was no one “sentinel” cell type sensitive to all toxins, which could be used to triage compounds for screening against additional cell types and assays.

A striking finding from the current study is the lack of similarity in the patterns of compound activity in cells derived from the same tissue but from different species (e.g., human HepG2 and rat H-4-II-E hepatoma cells). Even cells of similar tissue origin from the same species sometimes showed considerable differences in compound activity profiles. For example, the human SK-N-SH and SH-SY5Y lines showed quite different response patterns across the 1,353 compounds, even though SK-N-SH is the parental line of the SH-SY5Y. Overall, SH-SY5Y cells were more sensitive to compound-induced toxicity than SK-N-SH cells; the former cells are more differentiated and express a variety of neurotransmitters and neuronal cell surface markers. Neuronal cells are sensitive to environmental insults, perhaps because of their high metabolic demands and physiologic/morphologic specialization (Jellinger 2006). In general, rodent cells were more sensitive than the human cells in this study, and in each case of homologous cells from the two species, rodent cells were the more sensitive cell type. Cell doubling times did not correlate with sensitivity (as measured by the number of positive compounds), but future studies will investigate other possible mechanisms for the differential toxicities observed. In all, these studies demonstrate that *in vitro* cytotoxicity is often cell-type specific and that cytotoxicity in one cell type does not necessarily predict cytotoxicity in another.

One important limitation of the assay used here is that cytotoxicity was measured at a single time point (40 hr) only. To address this limitation, dynamic measurements of cellular response to exposure over time were made with selected compounds and the time-courses of cytotoxicity correlated with known molecular and cellular mechanisms of compound activity. Although this system is low throughput, staged screening of all compounds in the end point cytotoxicity assay followed by selective testing in the dynamic system may allow inference of mechanism of cytotoxicity.

In this study we have shown that it is feasible to screen large numbers of compounds in a titration-based HTS format and generate robust and reproducible results that can be analyzed to detect and compare cytotoxicity of a large number of compounds rapidly in a variety of cell types. To facilitate the use of these data by others, we have deposited all data from this study into a public database (PubChem 2007b) in advance of publication, and we will continue to do so as more compounds, conditions, cell types, and assay readouts are tested. Large qHTS data sets promise to provide a rich source of information for the development of *in vitro* toxicologic profiles that may prove valuable for prioritizing compounds for more intensive toxicologic investigation, and ultimately, predicting *in vivo* toxicity.

## Figures and Tables

**Table 1:** Cell types tested.

Species	Cell type	Origin	Status^a^	Doubling time (hr)
Human	HEK293	Embryonic kidney cells	T	28
Human	HepG2	Hepatocellular carcinoma	T	37
Human	SH-SY5Y	Neuroblastoma	T	61
Human	SK-N-SH	Neuroblastoma	T	56
Human	Jurkat	T-cell leukemia	T	22
Human	BJ	Foreskin fibroblasts	NT	147
Human	HUV-EC-C	Vascular endothelial cells	NT	148
Human	MRC-5	Lung fibroblasts	NT	132
Human	Mesangial	Renal glomeruli	NT	22
Rat	Proximal tubules	Cells from kidney	P	ND
Rat	H-4-II-E	Hepatoma	T	22
Mouse	N2a	Neuroblastoma	T	25
Mouse	NIH 3T3	Fibroblasts, embryonic	NT	28

Abbreviations: ND, not determined; NT, nontransformed; P, primary; T, transformed.

**Table 2:** Sensitivity (mean ± SD) of the 13 cell types to the tamoxifen positive control.

Cell type	*R*^2^	Hill coefficient	IC_50_ (μM)	CV	Z’ factor	S/B
Jurkat	0.98 ± 0.00	2.0 ± 0.2	8 ± 1	10.0 ± 8.7	0.86 ± 0.06	83 ± 10
HepG2	0.96 ± 0.02	4.4 ± 0.5	43 ± 8	8.8 ± 5.7	0.87 ± 0.03	25 ± 1
Rat kidney proximal tubule	0.96 ± 0.01	4.7 ± 0.3	44 ± 5	7.6 ± 5.1	0.88 ± 0.02	22 ± 1
HUV-EC-C	0.95 ± 0.02	4.2 ± 0.5	46 ± 5	8.9 ± 4.3	0.82 ± 0.07	26 ± 1
SK-N-SH	0.92 ± 0.02	3.4 ± 0.7	57 ± 13	10.3 ± 5.7	0.84 ± 0.06	27 ± 1
H-4-II-E	0.93 ± 0.02	2.7 ± 0.8	63 ± 15	8.5 ± 7.3	0.91 ± 0.02	25 ± 1
SH-SY5Y	0.91 ± 0.03	3.1 ± 0.7	65 ± 15	12.4 ± 7.1	0.71 ± 0.06	12 ± 3
MRC-5	0.90 ± 0.05	2.9 ± 1.2	68 ± 17	9.3 ± 4.7	0.83 ± 0.07	26 ± 1
N2a	0.95 ± 0.05	4.0 ± 0.8	72 ± 13	8.6 ± 6.7	0.89 ± 0.05	25 ± 1
NIH 3T3	0.91 ± 0.02	2.6 ± 0.8	79 ± 4	9.7 ± 6.5	0.44 ± 0.10	4 ± 1
HEK293	0.91 ± 0.04	3.8 ± 1.0	79 ± 10	9.8 ± 5.1	0.84 ± 0.08	25 ± 1
BJ	0.89 ± 0.04	3.1 ± 1.0	79 ± 10	10.4 ± 4.6	0.80 ± 0.08	20 ± 4
Mesangial	0.85 ± 0.03	2.4 ± 0.8	79 ± 10	6.9 ± 4.6	0.91 ± 0.03	25 ± 1

S/B, signal-to-background ratio.

**Table 3:** Curve classification and potency distribution of the NTP 1,408 compounds in rat primary kidney proximal tubule cells.

	Curve classification^a^					
IC_50_ range	1a	1b	2a	2b	3	4
< 100 nM (%)	1 (0.07)	2 (0.1)	0	0	0	
1 μM–100 nM (%)	5 (0.4)	0	0	0	0	
10 μM–1 μM (%)	10 (0.7)	0	0	4 (0.3)	0	
80 μM–10 μM (%)	23 (1.6)	7 (0.5)	30 (2.1)	71 (5.0)	21 (1.5)	
> 92 μM (%)						1,233 (87.6)

a See “Materials and Methods” for curve class definitions.
